# Oxidative Stress in Down and Williams-Beuren Syndromes: An Overview

**DOI:** 10.3390/molecules26113139

**Published:** 2021-05-24

**Authors:** Marta Ferrari, Stefano Stagi

**Affiliations:** Department of Health Sciences, University of Florence, Anna Meyer Children’s University Hospital, 50139 Florence, Italy; stefano.stagi@unifi.it

**Keywords:** oxidative stress, Down syndrome, Williams-Beuren syndrome

## Abstract

Oxidative stress is the result of an imbalance in the redox state in a cell or a tissue. When the production of free radicals, which are physiologically essential for signaling, exceeds the antioxidant capability, pathological outcomes including oxidative damage to macromolecules, aberrant signaling, and inflammation can occur. Down syndrome (DS) and Williams-Beuren syndrome (WBS) are well-known and common genetic conditions with multi-systemic involvement. Their etiology is linked to oxidative stress with important causative genes, such as *SOD-1* and *NCF-1*, respectively, of the diseases being primarily involved in the regulation of the redox state. Early aging, dementia, autoimmunity, and chronic inflammation are some of the main characteristics of these conditions that can be associated with oxidative stress. In recent decades, there has been a growing interest in the possible role of oxidative stress and inflammation in the pathology of these conditions. However, at present, few studies have investigated these correlations. We provide an overview of the current literature concerning the role of oxidative stress and oxidative damage in genetic syndromes with a focus on Down syndrome and WBS. We hope to provide new insights to improve the management of complications related to these diseases.

## 1. Introduction

The concept of oxidative stress (OS) was first introduced in 1985 [1,2]. Since then, it has been extensively investigated in different fields of biology and medicine. OS is the result of an imbalance in the redox state of a cell or a tissue, either because of an overproduction of reactive oxygen species and nitrogen (ROS/RNS) or a decreased antioxidant response [1,3,4]. ROS are a physiological consequence of the aerobic metabolism of a cell and are fundamental for intracellular signaling, transcription, and regulation of genes. The mitochondrial electron transport chain (ETC), involved in oxidative phosphorylation, is thought to be the main intracellular source of ROS generation. Similarly, several oxidoreductases located on the mitochondrial membrane are able to contribute to ROS production [5]. As a consequence, any change in the structure of mitochondria can result in increased ROS production [6,7]. Moreover, a relevant amount of ROS is generated by membrane-bound NADPH oxidase enzymes (NOX) [8]. This aspect is of fundamental importance in relation to some genetic syndromes in which genes involved in the regulatory pathways of these molecules are mutated [9]. Other intracellular organelles such as peroxisomes, lysosomes, and the endoplasmic reticulum contain enzymes that can contribute to cellular ROS production [10].

Chronic exposure to high levels of ROS induces biomolecule and signaling alterations leading to pathological events such as inflammation, early senescence, and apoptosis [2,3]. Many of these mechanisms are thought to play a pivotal role in atherosclerosis, cancer, diabetes, cardiovascular diseases, ageing, and neurodegenerative diseases [2,3,11]. In recent years, evidence has accumulated, but many questions still need answers. The study of genetic models of oxidative stress and genetic syndromes with mutations of genes involved in the regulation of oxidative stress can lead to new insights. Genetic syndromes are useful models for studying the function of a particular gene or process. Over the years, a broad range of genetic diseases have been investigated for the implications of OS and mitochondrial disfunction in their pathogenesis. Many of these fall within the category of cancer-prone and/or early ageing diseases such as Ataxia-Telangiectasia, Fanconi anemia, Werner syndrome, and Hutchinson-Gilford syndrome [12,13]. 

Down syndrome (DS) and Williams-Beuren syndrome (WBS) are two well-known conditions in which chromosomal alteration affects genes involved in the regulation of the redox state, such as Cu/Zn superoxide dismutase 1 (*SOD-1*) and neutrophil cytosolic factor 1 (*NCF-1*), respectively. In both syndromes, many clinical features such as early ageing and onset of cardiovascular and neurodegenerative diseases are thought to be a consequence of oxidative stress damage. In recent years, the life expectancy of these syndromes has significantly improved. From the study of these in vivo models, it may be possible to gain insight into other diseases that are much more common in the general population, such as dementia and Alzheimer’s disease. 

We aimed to review the current literature concerning the role of oxidative stress and oxidative damage in DS and WBS, with the dual intent of broadening knowledge about genetic syndromes and providing possible insights into the role of oxidative stress in other diseases.

## 2. Down Syndrome

Down syndrome (DS; OMIM #190685) is a complex genetic medical condition associated with trisomy 21, responsible for altered development during embryogenesis and organogenesis. Compared with other syndromes with a genetic etiology related to a redox imbalance, DS has been extensively studied [14]. Many of its clinical features such as accelerated ageing, neurological and cognitive impairment, congenital and acquired cardiovascular disease, immune disorders, thyroid problems, and coeliac disease have been investigated as possible consequences of cellular senescence and oxidative stress [14,15]. Several studies performed on animal and human models of trisomy 21 have shown a pro-oxidant status and an increased susceptibility to oxidative damage [16,17,18]. However, establishing the exact correlation between the clinical phenotype and the gene dosage effect of chromosome 21 may represent only a small part of a much larger situation [19]. Given the complexity of the clinical manifestations, the abnormal expression of genes located on chromosome 21, in association with responses to environmental stimuli, might also alter the expression of disomic genes. Many studies have demonstrated that a dysregulation of the genes and proteins involved in mitochondrial pathways, ATP consumption, and increased production of ROS may explain the wide range of phenotypes [6,20].

## 3. Role of Oxidative Stress in DS

Damage induced by excessive oxidative stress is an early onset, age-related process that accompanies DS patients throughout their lives. Premature ageing and neurodegenerative alterations are common clinical features in these patients. 

Altered mitochondrial activity and oxidative stress have long been associated with DS [16,21,22]. Modifications in the mitochondrial morphology and structure have been demonstrated in different DS tissues and cells [23,24]. For example, studies performed on human fetuses demonstrated that, compared with euploid cells, DS fibroblasts show significant damage in the structure of the mitochondria with an abnormal mitochondrial cristae morphology and an impairment in mitochondrial respiratory activity. These modifications are more evident in fetuses displaying heart defects, suggesting that mitochondrial dysfunction might be associated with a more severe phenotype [23]. Several mitochondrial DNA mutations; decreased mitochondrial membrane potential; broken, shorter, or highly swollen cristae; and macroscopic alteration of mitochondrial morphology are evident in cultures of astrocytes, neurons, and fetal fibroblasts from DS patients [6,23,24,25]. 

**Figure 1:**
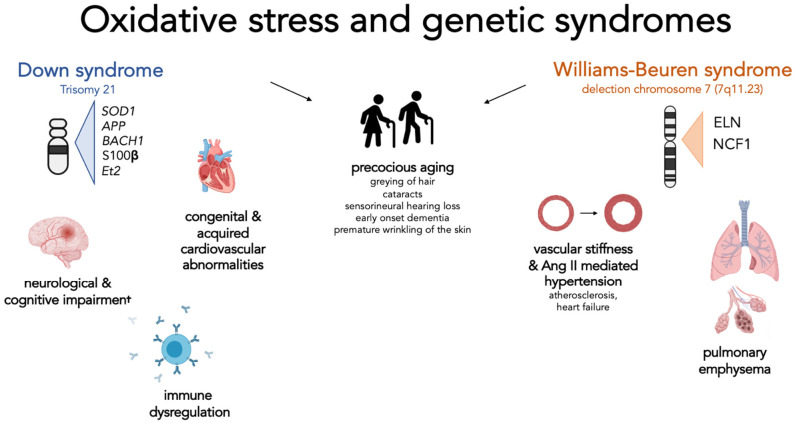
Oxidative stress and genetic syndrome, a focus on Down and Williams-Beuren syndromes. This figure summarizes for each syndrome the genes involved in the regulation of oxidative stress and the clinical manifestations that have been attributed to the effect of excessive oxidative stress. Interestingly, for both syndromes, a possible role of the indicated genes in directly or indirectly causing signs of premature ageing has been hypothesized. **Down syndrome—Cognitive and neurological disorders:** represent one of the main features of these patients: the overexpression of SOD1 and APP is associated with mitochondrial alterations, increased oxidative damage on neuronal cells, and early formation of senile plaques. Elevated expression of Ets-2 is associated with increased neuronal apoptosis. An abnormal production of BACH1 increases the risk of neurodegeneration. S100β, when overexpressed, leads to increased production of ROS. **Immune dysregulation:** SOD1 plays a role in modifying the activity and response of T cells; **Congenital and acquired cardiovascular abnormalities:** although no specific gene has been identified, it seems that increased oxidative stress and mitochondrial dysfunction are associated with increased development of these complications. **Williams-Beuren syndrome—vascular stiffness and Ang-II-mediated hypertension:** Elastin haploinsufficiency is associated with the presence of increased vessel thickness and stiffness resulting in reduced lumen and increased pressure. These changes result in a physiological increase in angiotensin II and activation of NADPH. The subsequent level of oxidative stress and the response of the cell to the effect of angiotensin II depend on the number of copies of the NCF1 gene involved by the deletion; **Pulmonary emphysema:** Elastin haploinsufficiency predispose subjects to premature development of pulmonary emphysema.

Several genes encoded on chromosome 21 such as *SOD1*, *APP*, *BACH1*, *Et2*, and *S100B* are thought to be involved in the increased OS levels found in DS mouse models and individuals [7,20] (Figure 1, Table 1). 

The human Cu/Zn superoxide dismutase 1 gene (*SOD-1*; OMIM *147,450) was the first chromosome 21 gene to be characterized and identified in different DS tissues [26]. It is one of the major enzymes involved in antioxidant defense, and catalyzes the dismutation of superoxide anion (O_2_^−^) to molecular oxygen and hydrogen peroxide (H_2_O_2_), which in turn is metabolized by catalase (CAT) and glutathione peroxidase (GPX) to water [27]. SOD-1 has been found at levels approximately 50% higher than normal in a variety of DS cells and tissues, including erythrocytes, B and T lymphocytes, and fibroblasts [7]. In the brain, the overexpression of *SOD-1* is combined with a physiological reduction in CAT and GOX; therefore, in the DS brain there is often an imbalance in the ratio of *SOD-1* to CAT and GPX, which leads to an accumulation of H_2_O_2_ and consequent damage [28]. Studies performed on mice overexpressing wild-type human *SOD-1* (Tg-SOD1) and brain cell cultures have demonstrated that exposure to ROS results in modifications that occur very early during neuronal development with vacuolization, membrane disruption and swelling, and rapid induction of apoptosis [29,30]. It is possible that the damage observed in fetal cells is due not only to the over-expression of *SOD-1*, but also to the lack of antioxidant enzymes, such as glutathione transferases and thioredoxin peroxidases [31]. Some authors have proposed the use of *SOD-1* and other genes located on chromosome 21 as possible biomarkers for DS in the context of non-invasive screening [32,33]. 

The spectrum of functions in which *SOD-1* is involved is extremely wide and not limited to redox cell balance. There is compelling evidence that the enzyme is involved in immune regulation through a relationship with T-cell activation and response [10,34], although no specific study has yet been carried out on DS patients. Defining the role of *SOD-1* can help to shed light on the state of immune dysregulation that characterizes patients with DS.

Among the protein-coding genes located on chromosome 21, the APP gene (amyloid beta A4 precursor protein; OMIM *104,760) deserves special attention. It was first identified and isolated on chromosome 21 in 1987 [35], and encodes for a precursor protein of beta-amyloid (Aβ), the main component of senile plaques, and one of the neuropathological findings of Alzheimer’s disease.

The study of this gene in subjects with DS has contributed to the development of the amyloid cascade hypothesis [36]: an explanation for dementia occurring in patients with Alzheimer’s disease. DS is the most common cause of early onset Alzheimer’s disease-dementia (AD-DS) [19,37], and approximately two-thirds of individuals with DS develop a type of dementia by the age of 60 years [38,39].

In individuals with DS, increased APP expression is strongly associated with Aβ deposition in adult life, and early and increased formation of senile plaques [7,14,38,40,41]. Oxidative stress and early plaque formation in the brain are closely connected. ROS damage increases the likelihood of the formation of protein aggregates as it interferes with the normal processes of protein elimination. Aβ aggregates can be targets of oxidative processes, inserting as oligomers within the cell membrane and promoting a process of lipid peroxidation (LPO). Studies performed on aged DS brains have demonstrated that plaques containing a significant amount of oxidized Aβ are mainly distributed within microglia [42,43]. In this context, oxidatively modified Aβ may reflect the efforts of microglial cells to take up and degrade Aβ, and oxidative modification of Aβ may be interpreted as an early event in the Aβ pathogenesis of senile plaques [42,43,44].

It seems that increased APP expression causes a progressive accumulation of transmembrane-arrested APP, which in turn disturbs mitochondrial integrity, which ultimately results in impairment of energy metabolism [37,45]. These findings led to the hypothesis that overexpression of APP may promote mitochondrial dysfunction in DS independent of aberrant Aβ deposition [37,45].

Insights regarding oxidative damage in the brain have been reported from the analysis of two genes, S100β and Ets-2, both located on chromosome 21.

S100β (S100 calcium-binding protein, beta; OMIM *176,990) is an astroglia-derived Ca^2+^-binding protein actively secreted from astrocytes that modulates the activity of neurons, microglia, astrocytes, monocytes, and endothelial cells [46]. S100β triggers trophic or toxic effects on neurons, depending on its concentration. Its expression levels are increased in both DS and AD astrocytes in association with neuritic plaques [47]. Studies performed on human DS neural progenitor cells showed that S100 β is constitutively overexpressed, inducing increased ROS formation and activation of stress response kinases [48]. Administration of S100B to the same cells resulted in a dose- and time-dependent ROS production and lipid peroxidation [48].

Studies on middle-aged DS patients have confirmed that chronic overexpression of S100β contributes to increased neuronal and neuritic APP expression with consequent accelerated amyloid deposition, as well as abnormal growth of neurites in β-amyloid plaques [37,49].

Ets-2 (ETS proto-oncogene 2, transcription factor; OMIM *164,740) is a member of the Ets family of transcription factors that has important functions in cancer, bone development, and immune responses [50]. Studies performed on DS cortical neurons demonstrated that elevated expression of Ets-2 is associated with increased neuronal apoptosis [51]. Most importantly, an increase in Ets-2 expression is induced by low concentrations of H_2_O_2_ and hyperexpression of enzymes SOD-1/GPX-1, both common characteristics of DS cells [37,52].

Another interesting transcription factor encoded on chromosome 21 is BACH1 (BTB domain and CNC homolog 1; OMIM *602,751), a transcription repressor that binds antioxidant response elements of DNA, thus inhibiting the transcription of specific genes involved in the cell stress response such as NADPH and heme oxygenase-1 (HO-1) [53]. The pivotal role of BACH1 in the OS response was demonstrated in mouse models. BACH1-deficient mice are more resistant to oxidative stress, and BACH1 overexpression enhances the production of reactive oxygen species (ROS) from the mitochondria of endothelial cells [53].

Given its role and location on chromosome 21, some authors investigated the possible role of BACH1 in patients with DS. Studies performed on mouse models and patients with DS demonstrated that BACH1 is significantly overexpressed compared with controls [54]. Dysregulation of the BACH1/OH-1 axis appears early and can, in association with other alterations, contribute to the early onset of neurodegeneration that characterizes DS patients. It is likely that upregulation of BACH1 due to trisomy 21 blocks the induction of antioxidant genes, thereby promoting an OS increase in the cell [7,20].

Lysosomal dysfunction has been increasingly recognized as a pathogenic factor in aging-related neurodegenerative disease. Recent studies have focused on possible correlations between chromosome 21 genes and lysosomal activities. Jiang et al. showed that modest elevations of endogenous APP in DS were sufficient to induce lysosomal disruption [55]. The gene impairs lysosomal acidification, depressing lysosomal hydrolytic activities and turnover of autophagic and endocytic substrates, processes that are vital to neuronal survival. These deficits, which are reversible by correcting lysosomal pH, are mediated by elevated levels of endogenously cleaved carboxy-terminal fragment of APP (APP-CTF). Similar endosomal-lysosomal pathobiology emerges early in sporadic AD, where neuronal APP-CTF is also elevated. This underscores its importance as a therapeutic target and the functional and pathogenic interrelationships between the endosomal-lysosomal pathway and genes causing AD. Possible additional influences of other triplicated chromosome 21 genes or metabolic factors, such as oxidative stress, lipid peroxidation, and protein nitration, may be considered likely additive factors in corrupting lysosome functions [20,55]. Taken together, these data illustrate the complexity of factors involved in the pathogenesis of OS in patients with DS [56]. Although the role of OS in some disease phenotypes is now evident, the exact mechanisms through which oxidative damage translates into the clinical features of DS need to be clarified. It is becoming increasingly evident that not only overexpression of some chromosome 21 genes, but also a more complex dysregulation of gene and protein expression associated with the trisomy may explain the main DS features [6].

## 4. Williams-Beuren Syndrome (WBS)

Williams-Beuren Syndrome (WBS), also known as Williams’ syndrome, is a rare genetic disorder with multisystemic involvement, affecting nearly 1 out of 7500–10,000 people. The extent of medical and developmental problems in patients with WBS is highly variable. Facial dysmorphisms, cardiovascular malformations, endocrinological alterations, and intellectual and cognitive disturbances represent common features of this syndrome [57]. WBS is caused by the deletion of a group of genes, approximately 25–28, located on chromosome 7 (7q11.23), including the ELN (elastin; OMIM *130,160) gene, which encodes for elastin, whose deletion is responsible for the cardiovascular hallmarks and accelerated ageing in patients with this syndrome [57,58,59]. Individuals with WBS often present signs of mildly accelerated ageing [59], such as the greying of hair during adolescence or young adulthood [60], cataracts, senile emphysema [61], high-frequency sensorineural hearing loss [62], premature wrinkling of the skin [63], and a precipitous age-associated decrease in long-term, episodic memory [64].

## 5. Role of Oxidative Stress in WBS

The correlation between ROS and clinical features in WBS is more difficult to clarify than in DS. There is evidence suggesting a possible pathophysiological role of increased OS in cardiovascular disease (hypertension, atherosclerosis, and heart failure) and abnormalities (mainly aortic stenosis). Approximately half of WBS patients develop hypertension [59,65,66].

Elastin alterations and deficiency occurring in WBS may play a pivotal role in making WBS patients predisposed to hypertension. Adult elastin knockout mice (Eln+/−) have smaller and thinner arteries, increased vessel stiffness, and hypertension that normalizes when the re-expression of elastin is restored [67]. Elastin-deficient mouse models often have elevated plasma renin and angiotensin II (Ang II) levels [67]. Many of the cellular actions of Ang II are mediated by the activation of the NADPH—oxidase (NOX) with the stimulation and formation of reactive oxygen species (ROS) [9]. Higher oxidative stress in elastin-insufficient vessels has been correlated with overexpression of a gene located on chromosome 7 named Ncf1 (neutrophil cytosolic factor 1; OMIM *608,512) [68]. The Ncf1 gene belongs to a group of 25–28 genes located on the part of chromosome 7 deleted in patients with WBS. Ncf1 encodes for p47phox, a protein acting as a regulatory subunit for several NOX family members that are expressed in the vasculature, which contribute to the production of ROS [69,70].

Differences in the WBS deletion affect the copy number for Ncf1, ultimately affecting hypertension risk and the severity of vascular stiffness [9,71]. Studies performed in Ncf1 knock-out mice revealed that p47phox has a major influence on Ang II [72] and that Ang-II-mediated oxidative stress in the vasculature may be the mechanism behind the protective effect in patients whose deletion includes a copy of Ncf1 [71,73,74]. Campuzano et al. demonstrated in a mouse model that the use of therapies with Ang II type 1 receptor blocker, losartan, or NOX inhibitor apocynin were effective in reducing the risk of hypertension [73]. As in humans, Ncf1 is likely to impact blood pressure in this model.

Haploinsufficiency at the elastin locus may predispose WBS subjects to premature development of pulmonary emphysema. This hypothesis is supported by murine models with a heterozygous deletion of the elastin gene (Eln +/−). While mice homozygous for the elastin gene deletion (Eln −/−) develop extensive changes in baseline lung architecture, heterozygous mice (Eln +/−) exhibit normal lung development and morphology despite having only approximately 45% of the lung elastin levels of wild-type mice [75]. However, when compared with wild-type (Eln +/+) mice, heterozygous mice exhibit an augmented inflammatory response and are more susceptible to developing emphysema when exposed to cigarette smoke. In the absence of a smoking history, other possible factors contributing to emphysema susceptibility may exist [75].

Recent studies hypothesized that mitochondrial dysfunction plays a role in WBS pathogenesis. In WBS-derived primary fibroblasts, decreased basal respiration and maximal respiratory capacity was found, as well as increased ROS generation and decreased ATP synthesis [76]. This mitochondrial dysfunction may be due to the loss of DNAJC30 (DNAJ/HSP40 homolog, subfamily c, member 30; OMIM *618,202), a gene included in the WBSCR. DNAJC30 knock-out mice showed reduced ATP levels as well as alterations in mitochondrial function [71].

Few studies have been published on the potential use of target therapies against oxidative stress in patients with WBS [71]. The complexity of elastin fiber assembly creates challenges for the search for possible therapies [77].

Given the high morbidity from cardiovascular complications and the intrinsic characteristics of elastin fibers, it is important to explore possibilities for developing new therapeutic strategies for WBS. Identifying the genes that modify the condition’s pathology could be helpful in this endeavor.

## 6. Conclusions

The chronic imbalance between free radical production and the pro-oxidative state within the cell determines a biological state defined as oxidative stress [1,3,4]. This condition seems to play a role in several diseases, although the exact mechanisms are still poorly understood [2,3,11].

Possible insights may be derived from the study of genetic syndromes such as DS and WBS, in which the chromosomal alteration affects OS-regulating genes [9,16,21,25]. Despite the accumulation of significant evidence, it has proved difficult to identify possible therapeutic targets. Given the close correlation between OS and mitochondrial function, the use of mitochondrial target therapies could be a strategy.

Some authors have proposed the use of mitochondrial nutrients, a group of agents that can directly or indirectly protect mitochondria from oxidative stress and increase their physiological function [78]. Some examples are acetyl-L carnitine (ALC), alpha-lipoic acid (ALA), and coenzyme Q10 (CoQ10); CoQ10 is one of the most studied in relation to DS. From a biochemical point of view, it represents an electron acceptor that transfers electrons from Complexes I and II to Complex III, it exerts an antioxidant function in lipid and mitochondrial membranes, and may also decrease electron leak from the electron transport chain. Because patients with DS appear to have lower CoQ10 values than healthy subjects [79], several clinical trials have focused on testing this mitochondrial nutrient as a possible therapy to reduce oxidative stress [80,81]. However, there have been no significant clinical improvements in patients treated with coenzyme Q, making its use in these patients questionable. Further studies are needed, and more promising results may be obtained from the use of mitochondrial nutrient combinations. Another strategy can be the use of APP as a therapeutic target. Understanding how to reduce the effects of increased dosage of this protein and its products may provide important insights for the management and treatment not only of patients with DS, but also of those with AD. In this sense, several approaches can be hypothesized to reduce APP levels. One of the most promising is the use of posiphen, an acetyl-cholinesterase inhibitor that is able to reduce in vivo and in vitro levels of APP and Aβ42 in neural cells. For its characteristics, posiphen is currently under study in patients with AD at early stages. Starting from these assumptions, Chen et al. investigated the role of this substance on the Ts65Dn mouse model of DS, confirming the data already obtained in vitro. More specifically, the oral use of posiphen, among other effects, normalized of the levels of fl-APP and its C-terminal fragments, and reduced the levels of Aβ42 [82]. This study is pivotal as it lays the foundation for possible use of this drug in patients with DS and AD.

Until specific treatments improving the clinical and biochemical prognosis in DS and WBS subjects are developed, we underline the importance of introducing antioxidant therapies combined with nutritional intervention to counteract the clinical damage induced by oxidative stress and improve cell metabolism. Future clinical studies with long-term follow-up on large cohorts of DS and WBS children precociously treated may clarify the influence of antioxidant therapies and diet on the clinical features of these syndromes.

## Figures and Tables

**Table 1:** Summary table of major altered genes by syndrome and their respective clinical consequences.

Scheme	Gene	Function	Modification and Clinical Consequences
Down syndrome (DS)	SOD-1 (*Cu/Zn superoxide dismutase 1*) (OMIM *147,450)	Antioxidant defense catalyzes the dismutation of superoxide radicals to hydrogen peroxide (H_2_O_2_), then metabolized to water by catalases or peroxidases.	Overexpression not accompanied by a corresponding increase in H_2_O_2_ handling enzymes, H_2_O_2_ excess that promotes oxidative damage by itself or leads to production of the noxious hydroxyl radical.
	APP (*Amyloid Beta A4 Precursor Protein*) (OMIM *104,760)	Encodes for a precursor protein of beta-amyloid (Aβ).	Overexpression—Aβ deposition and increased formation of senile plaques.
	BACH 1 (*BTB Domain And CNC Homolog 1*) (OMIM *602,751)	Transcription repressor inhibiting the transcription of specific genes involved in the cell stress response, such as NADPH and heme oxygenase-1 (HO-1)	Overexpression enhanced the production of reactive oxygen species (ROS) from the mitochondria of endothelial cells.
	S100 β (*S100 Calcium-Binding Protein, Beta*) (OMIM *176,990)	Ca^2+^-binding protein actively secreted from astrocytes modulating the activity of neurons, microglia, astrocytes, monocytes, and endothelial cells depending on its concentration	Overexpression induced increased ROS formation and contributed to increased neuronal and neuritic *APP* expression with consequent accelerated amyloid deposition.
	Et2 (*ETS Protooncogene 2, Transcription Factor*) (OMIM *164,740)	Transcription factor with important functions in cancer, bone development and immune responses.	Overexpression—associated with increased neuronal apoptosis and induced by low concentrations of H_2_O_2_ and hyperexpression of enzymes SOD-1/GPX-1, both common characteristics of DS cells.
Williams-Beuren syndrome (WBS)	ELN (*Elastin*) (OMIM *130,160)	Encodes for elastin, a key protein of the extracellular matrix in many tissues including vasculature, pulmonary tissue.	Haploinsufficiency—increased vessel stiffness, hypertension and premature development of pulmonary emphysema.
	NCF1 (*Neutrophil Cytosolic Factor 1*) (OMIM *608,512)	Encodes for p47phox, a protein acting as a regulatory subunit for several NOX family members expressed in the vasculature.	Overexpression affects Ang-II-mediated oxidative stress in the vasculature with higher oxidative stress in the elastin-insufficient vessels.

## Data Availability

Not applicable.

## Abbreviations

Ang II: angiotensin II; APP: amyloid beta A4 precursor protein; BACH1: BTB domain and CNC homolog 1; ELN: elastin; Et2: ETS protooncogene 2, transcription factor; NCF1: neutrophil cytosolic factor 1; SOD1: Cu/Zn superoxide dismutase 1; S100β: S100 calcium-binding protein, beta.
